# Aligner-mediated cleavage of nucleic acids and its application to isothermal exponential amplification†
†Electronic supplementary information (ESI) available: Experimental procedures, schematics, gel images, real-time fluorescence spectra and detailed nucleic sequence information. See DOI: 10.1039/c7sc05141g


**DOI:** 10.1039/c7sc05141g

**Published:** 2018-02-28

**Authors:** Wanghua Wu, Tao Zhang, Da Han, Hongliang Fan, Guizhi Zhu, Xiong Ding, Cuichen Wu, Mingxu You, Liping Qiu, Juan Li, Liqin Zhang, Xiang Lian, Rong Hu, Ying Mu, Jianguang Zhou, Weihong Tan

**Affiliations:** a Research Center for Analytical Instrumentation , Institute of Cyber-Systems and Control , State Key Laboratory of Industrial Control Technology , Zhejiang University , Hangzhou 310027 , China . Email: zhtao@zju.edu.cn; b Center for Research at Bio/nano Interface , Department of Chemistry , Department of Physiology and Functional Genomics , Health Cancer Center , UF Genetics Institute and McKnight Brain Institute , University of Florida , Gainesville , Florida 32611-7200 , USA . Email: tan@chem.ufl.edu; c Molecular Science and Biomedicine Laboratory , State Key Laboratory of Chemo/Biosensing and Chemometrics , College of Chemistry and Chemical Engineering , College of Biology , Collaborative Innovation Center for Molecular Engineering for Theranostics , Hunan University , Changsha 410082 , China; d Department of Environmental Medicine , Institute of Hygiene , Zhejiang Academy of Medical Sciences , Hangzhou 310013 , China

## Abstract

We herein describe a simple and versatile approach to use conventional nicking endonuclease (NEase) for programmable sequence-specific cleavage of DNA, termed aligner-mediated cleavage (AMC), and its application to DNA isothermal exponential amplification (AMC-based strand displacement amplification, AMC-SDA). AMC uses a hairpin-shaped DNA aligner (DA) that contains a recognition site in its stem and two side arms complementary to target DNA. Thus, it enables the loading of an NEase on DA's stem, localization to a specific locus through hybridization of the side arms with target DNA, and cleavage thereof. By using just one NEase, it is easy to make a break at any specific locus and tune the cleavage site to the single-nucleotide scale. This capability also endows the proposed AMC-SDA with excellent universality, since the cleavage of target DNA, followed by a polymerase-catalyzed extension along a particular primer as a key step for initiating SDA, no longer relies on any special sequence. Moreover, this manner of initiation facilitates the adoption of 3′-terminated primers, thus making AMC-SDA highly sensitive and highly specific, as well as simple primer design.

## Abstract 2

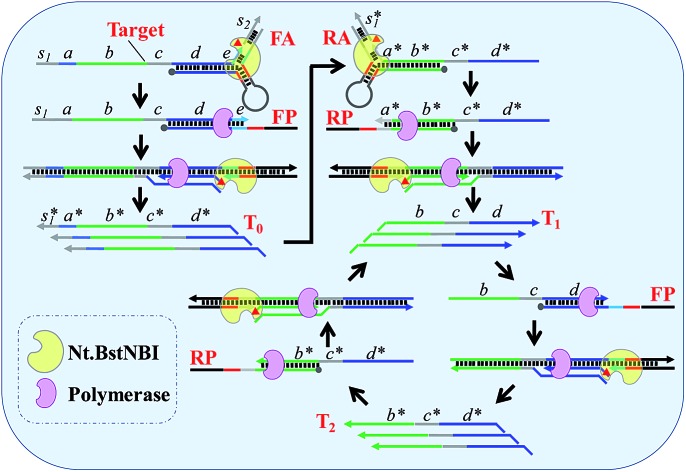
A programmable sequence-specific aligner-mediated cleavage endows strand displacement amplification with excellent universality, high sensitivity, high specificity and simple primer design.

## Introduction

Amplified detection of nucleic acids with high sensitivity and selectivity is important in many fields such as molecular biology, medical diagnostics and forensic analysis.^1^–^3^ At present, there are two main strategies for nucleic acid amplification: polymerase chain reaction (PCR) and isothermal amplification.^4^ While PCR is powerful and most widely used, the requirement for specialized thermal-cycling instrumentation and power supply makes it less suitable for on-site tests. As an alternative technique, isothermal amplification, which operates at a single optimal temperature, has relatively low hardware dependence, high amplification efficiency and short reaction time, and thus is a valuable tool in studies of nucleic acids, especially in terms of rapid tests and point-of-care diagnostics.^5^–^8^ Till now, a variety of isothermal amplification methods has been developed, such as loop-mediated amplification (LAMP),^9^,^10^ helicase-dependent amplification (HDA),^11^–^13^ rolling circle amplification (RCA),^14^–^17^ strand displacement amplification (SDA),^18^,^19^ smart amplification process (SMAP),^20^ recombinase polymerase amplification (RPA),^21^,^22^ cross priming amplification (CPA)^23^ and hybridization chain reaction (HCR).^24^–^26^ However, due to the challenges in using the newly synthesized double-stranded DNA as template for the next round of amplification, a crucial step for exponential reaction, most of the current isothermal methods employ sophisticated mechanisms or extra proteins/enzymes, which could cause complicated primer design or compromised performance.

Among others, SDA was one of the earliest methods developed in the 1990s.^18^,^27^ It uses a nicking endonuclease (NEase) to make a nick on one chain of a double-stranded DNA (dsDNA); then a strand-displacement polymerase catalyzes the extension from the nicking site. Due to this very simple mechanism, as well as its high amplification efficiency, SDA has been extensively studied in the past two decades.^28^–^32^ However, the use of endonuclease brings with it a major limitation in that the dsDNA must contain a recognition sequence. Thus, in exponential SDA, two head-to-head recognition sites are required, which seriously impedes universality. Such efforts as adoption of two extra bumper primers,^27^ fingerprinting techniques^33^ and beacon-assisted amplification^34^,^35^ are either unable to address this problem thoroughly, or are likely to worsen the nonspecific background amplification that is already serious in SDA. Therefore, it remains challenging to develop a versatile SDA method with high sensitivity and specificity.

**Scheme 1:**
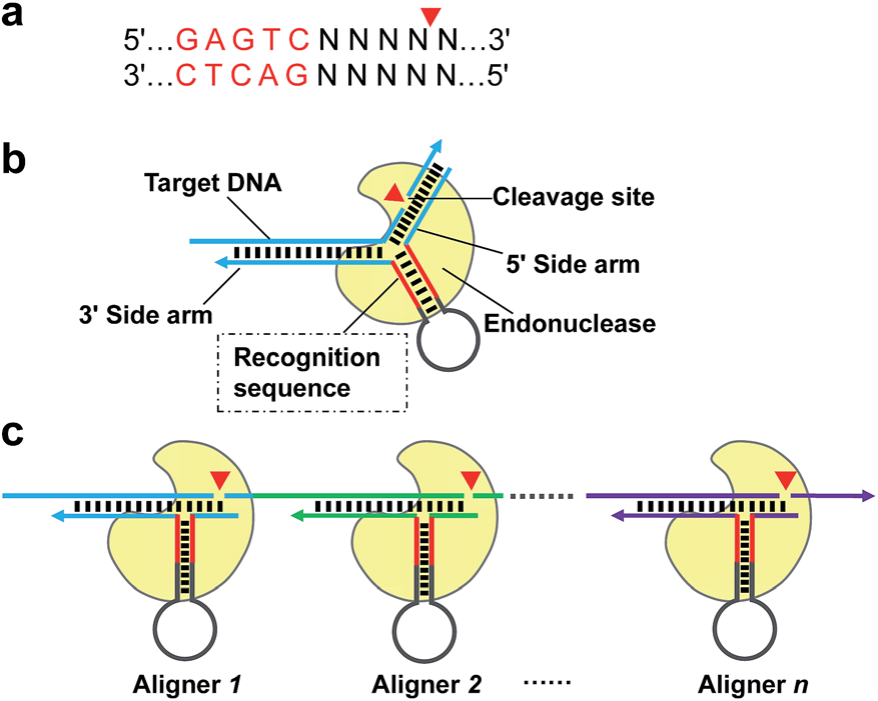
Schematic of AMC based on nicking endonuclease Nt.BstNBI. (a) Recognition sequence and cleavage site of Nt.BstNBI. (b) Basic structure of the proposed DNA aligner and working principle of AMC. Nt.BstNBI firstly binds to the recognition sequence in the stem of DNA aligner (Loading), and is aligned with a specific locus through the hybridization of the two side arms with target DNA (Localization), then cleaves at that site (Reaction). (c) Programmable, sequence-specific cleavage of DNA *via* AMC. By simply varying the sequences of the aligner's side arms, a break can be made at any locus of target DNA.

Given that the real challenge in SDA is the introduction of a recognition site of NEase onto both ends of the target DNA, a more straightforward procedure is to extend the target DNA along a particular primer to generate the sequence needed. However, this process must rely on specific cleavage of target DNA to precisely “redefine” its 3′ end. Therefore, in this paper, we first describe a novel strategy of using conventional NEase for programmable, sequence-specific cleavage of nucleic acids, called aligner-mediated cleavage (AMC). The working principle is based on a hairpin-shaped DNA probe having two components (Scheme 1b): a stem-loop structure with a recognition site for the NEase in the stem and two side arms complementary to the target sequence. As a result, the NEase can bind to the probe's stem and be localized to a specific locus, where cleavage is made *via* hybridization of the two side arms with target DNA. Note that the main function of the hairpin-shaped probe is to align the enzyme with a specific locus of target DNA, hence the term DNA aligner (DA). By simply modulating the sequence of the aligner's side arms, it is easy to align the NEase with any specific locus (Scheme 1c) and tune the cleavage site to the single-nucleotide scale. On this basis, an isothermal exponential amplification, AMC-SDA, has been developed. Because of the excellent versatility of AMC, the cleavage of target DNA, followed by extension along a particular primer to generate the recognition site of NEase, no longer relies on any special sequence. Thus, the proposed AMC-SDA is highly universal. In addition, as a result of adopting 3′-terminated aligners/primers, which can dramatically reduce the notorious nonspecific background amplification that commonly exists in most isothermal methods, AMC-SDA also features high sensitivity and specificity. In addition, primer design becomes easier, as well, because only two domains of target DNA need to be considered, with less concern about primer-dimer-related artifacts.

## Results and discussion

### Feasibility of AMC

**Fig. 1:**
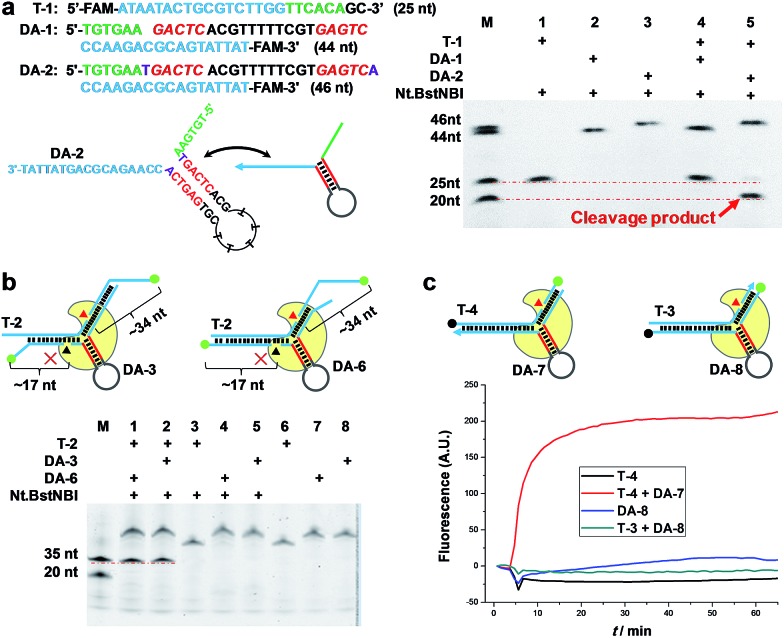
AMC makes a break exclusively on target DNA. (a) Representative sequence/structure of DNA aligner (Left). The denaturing PAGE image shows that only the DNA aligner with one extra base pair beyond the recognition site can induce cleavage (Right). (b) Schematics of DNA complexes formed by fluorescently labelled target DNA and aligners (Top). The denaturing PAGE image shows that aligner-mediated cleavage exclusively occurs on target DNA (Red triangle), rather than on DNA aligner (Black triangle) (Bottom). (c) Schematics of DNA complexes formed by dual-labelled (Dabcyl and FAM) aligner or target sequence (Top). Real-time fluorescence upon adding Nt.BstNBI at 55 °C also shows a break of target sequence but not DNA aligner (Bottom).

To demonstrate the feasibility of AMC, a nicking endonuclease, Nt.BstNBI, was first examined. Nt.BstNBI is a typical Type IIS endonuclease that cleaves only one strand of dsDNA at 4 bases downstream of the recognition site (Scheme 1a). By using two similar DAs, DA-1 and DA-2, which differ only in an extra A-T pair beyond the recognition site of DA-2 (Fig. 1a), a new, shorter band can only be found in the case of DA-2/T-1/Nt.BstNBI, along with the intact DA-2 band and the disappearance of target DNA T-1 (Fig. 1a). This clearly indicates the digestion of T-1, as well as the necessity of an extra base pair beyond the recognition site. It is likely that nucleotides right at the three-way junction cannot form stable Watson–Crick base pairs under current conditions, resulting in an incomplete recognition site. This can explain why DA-1 does not work. However, two or more base pairs beyond the recognition site will lead to the digestion of DA itself, either with or without target DNA (Fig. S1†). Therefore, an optimal design of our DNA aligner contains only one such base pair as shown in Fig. 1a.

To further understand the cleavage pattern of AMC and confirm whether the conformational changes of DNA structure have any effects on it, we devised four fluorescently labeled aligners, each having different hybridization length with target DNA in either the 3′ or 5′ side arms, with the aim of making the final complex more like an asymmetric Y-shaped structure, rather than a symmetric one. As shown in Fig. 1b and S2,† all cases resulted in a ∼34 nt band rather than a ∼17 nt band, indicating the cleavage of target DNA. Besides, the spectroscopic study also shows the break of target sequence instead of DA (Fig. 1c). These results confirm that the proposed DNA aligner mediates the cleavage by Nt.BstNBI exclusively on target DNA.

### Programmable, sequence-specific cleavage of DNA

**Fig. 2:**
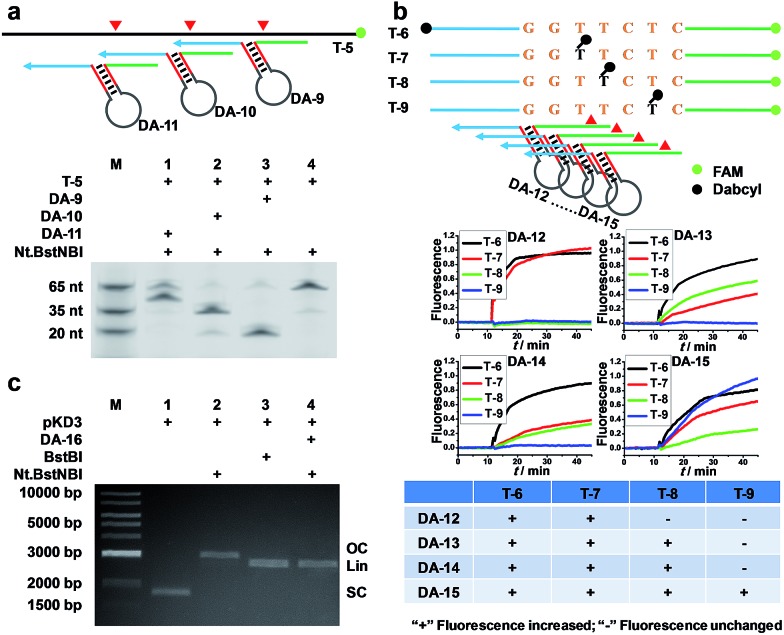
Programmable, sequence-specific cleavage of DNA *via* AMC. (a) Schematic (Top) and denaturing PAGE image (Bottom) showing that a target strand was cut at different sites by simply varying the sequences of the DNA aligner's side arms. (b) Fluorescence study showing that the cleavage site can be tuned down to one nucleotide. The schematic (Top) displays the labelling sites of FAM and Dabcyl, as well as the positions where DNA aligners bind. In a crossover trial, temporal fluorescence changes upon adding Nt.BstNBI at 55 °C were recorded (Middle), and then the patterns of fluorescence increase for each DNA aligner were summarized (Bottom). (c) Agarose gel image showing that a plasmid was cut *via* AMC. SC, OC and Lin represent super-coiled, open circular and linearized plasmid, respectively.

To verify that AMC is capable of sequence-specific cleavage of target DNA in a programmable manner, we designed three DAs that corresponded to different loci of a FAM-labeled target strand. As shown in Fig. 2a, three different bands of ∼20, ∼35 and ∼50 nucleotides were respectively produced under the guidance of these aligners. These results are very consistent with our expectations and confirm that the proposed DNA aligner is able to direct Nt.BstNBI to align with and cut at any specific locus of target DNA by simply modulating the sequence of its side arms. Further studies even showed that the cleavage site can be tuned by only one nucleotide. As illustrated in Fig. 2b, this experiment involved: (1) four target DNAs that have identical sequences with FAM at the 3′-end and Dabcyl at different sites; and (2) four DAs whose binding site on target DNA varies by one nucleotide in the series. When crossover trials were carried out, distinct patterns of fluorescence increase were obtained for each DA. Such patterns allowed us to easily infer the exact cleavage site, *i.e.*, always at 3 nucleotides downstream of the Y junction for all the four DAs, thus confirming the tunability at the single-nucleotide scale.

In another experiment, we also showed the cleavage of plasmid *pKD3* (Fig. S3†), indicating the extensive applicability of AMC. As seen in Fig. 2c, Nt.BstNBI itself nicked the plasmid into open circular format (Lane 2) owing to the existence of one GAGTC sequence in *pKD3*, while in the presence of a DNA aligner (DA-16, Table S1†), it linearized the plasmid (Lane 4) with a gel result similar to that of double-strand cleavage by endonuclease BstBI (Lane 3). In parallel, AMC functionality based on other endonucleases, such as Nt.AlwI, AlwI and Mlyl, was also demonstrated (Fig. S4†). These provide more choices for AMC-based cleavage of DNA and certainly endow this method with notable flexibility.

### AMC-based isothermal exponential amplification of DNA

**Scheme 2:**
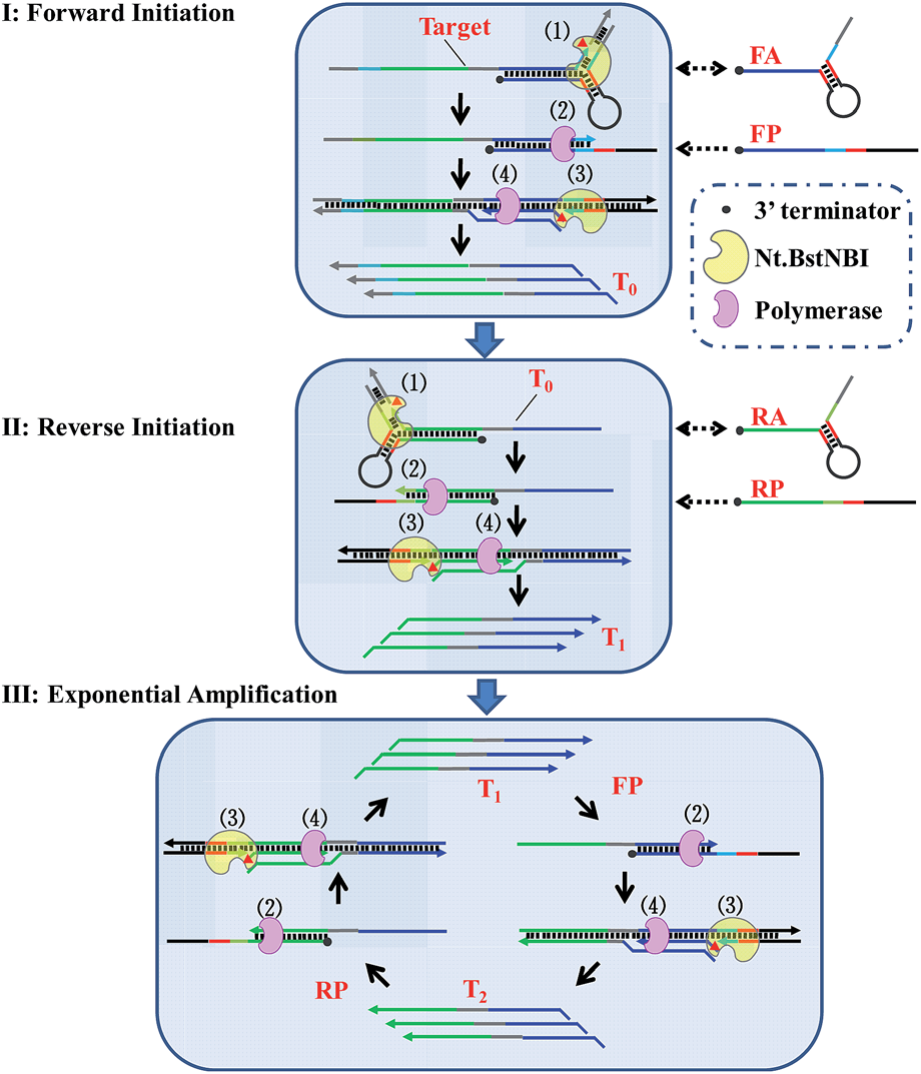
Schematic illustration of AMC-SDA. (Step 1) Target DNA, or its antisense sequence, hybridizes with DNA aligner and is cleaved by Nt.BstNBI through AMC. (Step 2) The cleaved sequence with a definite 3′-end binds a linear primer, followed by polymerase-catalyzed extension along this linear primer to generate a complete double-stranded recognition site. (Step 3) Nt.BstNBI binds to the newly formed recognition site and makes a nick four bases downstream. (Step 4) Polymerase catalyzes the extension from the nicking site, displacing the previous strand. Step 3 and Step 4 can be repeated many times.

By taking advantage of AMC's versatility, a universal method for isothermal exponential amplification of DNA, AMC-SDA, was successfully developed. As shown in Scheme 2 (see more details in Scheme S1†), the proposed AMC-SDA mainly consists of three stages: forward initiation, reverse initiation and exponential amplification. In forward initiation, a target DNA of interest first hybridizes with forward aligner (FA) and is cleaved by Nt.BstNBI through AMC (Step 1). Then, the cleaved target leaves FA, because the shortened hybridization length is not sufficient to maintain a stable Y-shaped structure. Instead, it binds a linear primer (FP) that contains the recognition sequence of Nt.BstNBI, followed by polymerase-catalyzed extension along FP to generate a complete double-stranded recognition site (Step 2). As a result, free Nt.BstNBI in the system is able to bind the newly formed recognition site and make a nick four bases downstream (Step 3), where a second polymerase-catalyzed extension will synthesize the antisense strand (T_0_) of target DNA (Step 4). Cycling Step 3 and Step 4 will result in more and more T_0_. Similarly, an identical process will occur in reverse initiation using T_0_ as template and RA/RP as reverse aligner/primer to generate the sense strand (T_1_). At this point, not only has the original target sequence been amplified linearly, but its 3′ and 5′ ends have been precisely redefined. This further promotes the reaction into the exponential amplification stage, wherein T_1_ hybridizes with primer FP at first, followed by Steps 2–4, as described above, to continuously generate the antisense strand T_2_. Afterwards, T_2_ and primer RP undergo a similar process to continuously regenerate T_1_. Iterations of these processes will accumulate more and more T_1_ and T_2_ as the final amplification products. It is noteworthy that AMC-SDA does not begin with the extension of primers along target DNA, as most other amplification methods do. Consequently, a 3′-terminator (*e.g.*, 3′ inverted-dT) can be applied. This prevents unexpected extensions of both primer and aligner along the target sequence, leading to inefficient amplification (Fig. S5†). In addition, the notorious nonspecific background amplification that commonly occurs in most isothermal methods^36^,^37^ can, to some extent, be restrained, thereby allowing us to characterize AMC-SDA as highly sensitive and specific, while, at the same time, affording easy primer design.

### Feasibility, sensitivity and specificity of AMC-SDA

**Fig. 3:**
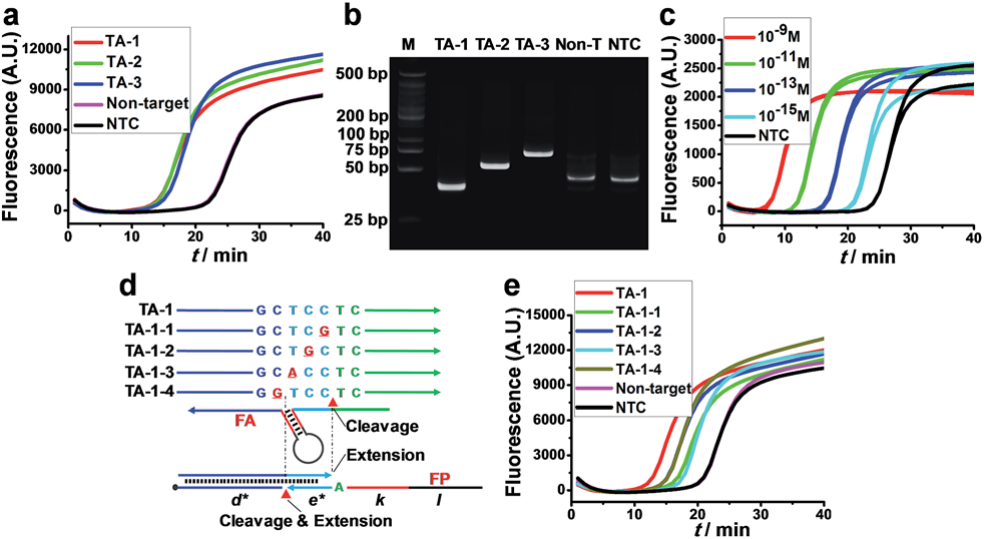
Feasibility, sensitivity and specificity of AMC-SDA. (a) Real-time fluorescence and (b) PAGE image for three target DNAs (10^–11^ M) of different lengths. (c) Real-time fluorescence caused by different concentrations of target DNA (TA-1). (d) Schematic showing the positions of mismatching and the sites where the cleavage/extension occurs in forward initiation. (e) Real-time fluorescence caused by matched and single-base mismatched sequences (10^–11^ M).

The feasibility of AMC-SDA was verified by conducting the amplification of three artificial sequences with different lengths (TA-1–TA-3). All cases showed a sigmoidal fluorescence curve (Fig. 3a) with a point of inflection (POI, defined as the time corresponding to the maximum slope) much lower than that of a nontarget sequence and H_2_O as no-target control (NTC). Meanwhile, the PAGE image showed neat bands of expected length (Fig. 3b). To further evaluate the sensitivity of AMC-SDA, real-time fluorescence of SYBR Green I caused by different concentrations of TA-1 was measured. Generally, target DNA as low as 1 fM can be detected (Fig. 3c). And the POI shows a linear correlation with log[DNA] (Fig. S6†). If the primers/aligners are of high quality, *i.e.*, efficient labeling of 3′-terminator and maintenance in solution, a much lower concentration down to 1 aM could be obtained (Fig. S7†). This verifies our expectations that the 3′-terminator not only restrains primer-dimer-related nonspecific amplification, but also prevents unexpected extensions of both aligner and primer along target DNA (Fig. S5†). When using a molecular beacon as reporter, we can even achieve ultrahigh sensitivity with zero background (Fig. S8†). Moreover, our method was also able to discriminate a single-base mutation near the cleavage site of AMC (Fig. 3d and e). We attribute this feature to a dual-identification process, *i.e.*, mismatches in this region will decrease the efficiency of both cleavage and extension steps. Therefore, it is reasonable that TA-1-1 and TA-1-3 caused the higher POI, since their mismatches are right at the cleavage sites, which are also the sites where polymerase-catalyzed extension begins (Fig. 3d).

### Universality and practicality of AMC-SDA

**Fig. 4:**
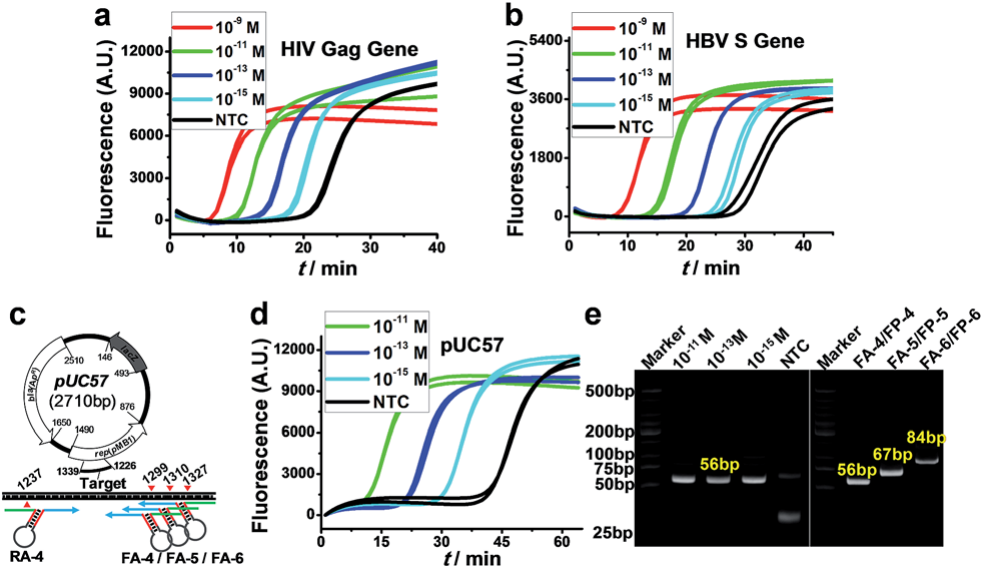
Universality of AMC-SDA. (a and b) Real-time fluorescence for various concentrations of target sequences from HIV Gag gene and HBV S gene, respectively. (c) Schematic of a target fragment in plasmid *pUC57* and the cleavage sites mediated by various forward/reverse aligners. (d) Real-time fluorescence for a 56-bp fragment from no. 1240 to 1296 base of *pUC57*. (e) PAGE image showing the amplification products corresponding to (d) (Left) and three fragments with different length from plasmid *pUC57* when using various forward aligners/primers (Right).

More importantly, because of the excellent versatility of AMC, the cleavage of target DNA, as a key step for initiating amplification, no longer relies on any special sequence; therefore, the proposed AMC-SDA is also highly universal. This was demonstrated by the amplification of two more sequences from the *HIV Gag gene*^38^ and *HBV S gene*,^39^ respectively, with both cases displaying the ability to detect 1 fM DNA (Fig. 4a and b). To further verify its practical utility, we also used AMC-SDA to amplify a 56-bp fragment of a plasmid (*pUC57*). It also showed a sensitivity of 1 fM (Fig. 4d) and the expected bands in the PAGE image (Fig. 4e). Besides, three fragments of different length (56 bp, 67 bp and 84 bp) from this plasmid were also examined using the same reverse aligner/primer pair, RA-4/RP-4, and varying forward counterparts, including FA-4/FP-4, FA-5/FP-5, and FA-6/FP-6 (Fig. 4c). As shown in Fig. 4e, all cases displayed the neat bands of expected length, again confirming the excellent universality and efficacy of AMC-SDA. To evaluate the practicality of AMC-SDA for real samples, the detection of HBV DNA in clinic serum specimens was performed. It showed that as few as 2.5 × 10^4^ copies of HBV DNA could be clearly discriminated from NTC (Fig. S9†). This performance is consistent with the sensitivity of our method (∼10^–15^ M in 25 μL volume). Furthermore, four HBV positive serum specimens and three HBV negative serum specimens were also tested. The results (Fig. S10a†) are well consistent with those obtained using a commercial HBV qPCR kits (Fig. S10b†), showing a good potential to detect the HBV DNA in real samples.

## Conclusions

We have developed a simple and versatile strategy, AMC, for programmable, sequence-specific cleavage of DNA. It uses a DNA aligner to enable the loading of NEase and localization to a specific site, followed by site-specific cleavage. AMC uses only one NEase and does not require any special sequence in target DNA. More importantly, it can make a break in a programmable way and tune the cleavage site to the single-nucleotide scale, showing the greatest simplicity and versatility. Given the large amount of Type IIS endonucleases, with four having been verified here, AMC can also be characterized by its flexibility and, hence, adaptability to a wide variety of applications. Herein, an AMC-based isothermal exponential amplification, AMC-SDA, has been demonstrated, which also features excellent universality, as well as high sensitivity, high specificity and easy primer design, with the capability to detect 1 fM or less DNA and to discriminate a single-nucleotide mutation. Given that the performance of AMC-SDA is dependent on the quality of primers, further studies, *e.g.*, the adoption of phosphorothioated primers and introduction of analogous nucleotides (*e.g.*, locked nucleic acids, abasic sites, 2′-O-Me RNAs) that are neither templates nor substrates of polymerase, are underway, which are expected to endow this method with higher sensitivity, specificity and reproducibility.

## Conflicts of interest

There are no conflicts to declare.

## Supplementary Material

Supplementary information
