# Supplementary material for: Aligner-mediated cleavage of nucleic acids and its application to isothermal exponential amplification
Source: Chem Sci. 2018 Feb 28;9(11):3050–5. doi: 10.1039/c7sc05141g (PMC5916018; doi:10.1039/c7sc05141g)
Supplement: Supplementary file 1 [file SC-009-C7SC05141G-s001.pdf]

Electronic Supplementary Material (ESI) for Chemical Science.
This journal is © The Royal Society of Chemistry 2018

Aligner-Mediated Cleavage of Nucleic Acids and its Application to Isothermal Exponential Amplification

Wanghua Wu,[a] Tao Zhang,[a,b] Da Han,[b,c] Hongliang Fan,[a,d] Guizhi Zhu,[b,c] Xiong Ding,[a] Cuichen Wu,[b,c] Mingxu You,[b] Liping Qiu,[b,c] Juan Li,[b] Liqin Zhang,[b,c] Xiang Lian,[a] Rong Hu,[b,c] Ying Mu,[a] Jianguang Zhou[a] and Weihong Tan* [b,c]*

[a]Research Center for Analytical Instrumentation, Institute of Cyber-Systems and Control, State Key Laboratory of Industrial Control Technology, Zhejiang University, Hangzhou 310027, China.
[b]Center for Research at Bio/nano Interface, Department of Chemistry and Department of Physiology and Functional Genomics, Health Cancer Center, UF Genetics Institute and McKnight Brain Institute, University of Florida, Gainesville, Florida 32611-7200, USA.
[c]Molecular Science and Biomedicine Laboratory, State Key Laboratory of Chemo/Biosensing and Chemometrics, College of Chemistry and Chemical Engineering, College of Biology, Collaborative Innovation Center for Molecular Engineering for Theranostics, Hunan University, Changsha 410082, China.
[d]Department of Environmental Medicine, Institute of Hygiene, Zhejiang Academy of Medical Sciences, Hangzhou 310013, China.

*To whom correspondence should be addressed. Email: zhtao@zju.edu.cn, tan@chem.ufl.edu.

Table of Contents

Experimental Procedures Page S3-S5

Supporting Scheme and Figures Page S6-S11

Tables Page S12-S14

References Page S15

Experimental Procedures

Materials. All oligonucleotides of HPLC grade were synthesized on an ABI 3400 DNA synthesizer or purchased from Sangon Biotech (Shanghai, China) Co., Ltd. The detailed information is listed in Table S1-2. Endonucleases Nt.BstNBI, AlwI, Nt.AlwI and MlyI (10,000 units/mL), Bst 2.0 WarmStart DNA polymerase (8,000 units/mL), dNTPs (10 mM), Low Molecular Weight DNA Ladder, 1 kb DNA Ladder, NEBuffer 3.1 and $MgCl_2$ solution (25 mM) were purchased from New England Biolabs (Ipswich, MA). SYBR Green I (10,000×) and plasmid *pUC57* (0.5 μg/μL) were obtained from Invitrogen (Carlsbad, CA) and Sangon Biotech (Shanghai, China) Co., Ltd, respectively. Plasmid *pKD3* (0.2 μg/μL) was purchased from YouBio (Changsha, China) Co., Ltd. DNA-grade water (DNase and Protease-free) and Tris-HCl buffer (1 M solution, pH 8.0) were purchased from Fisher Scientific (Pittsburgh, PA). Bovine serum albumin (BSA) and other frequently used reagents were purchased from Sigma-Aldrich (St. Louis, MO). The HBV positive serum standards (2×10^6, 2×10^5 IU ml^{-1}), HBV TaqMan qPCR diagnosis kit, four HBV positive serum specimens and three negative serum specimens were provided by the DAAN Gene Co., Ltd. of Sun Yat-sen University (Guangzhou, China). DNA was extracted from serum specimens using RNA/DNA Extraction/Purification Kits according to the manufacturer's instructions.

DNA synthesis on the ABI 3400 DNA synthesizer. Solid-state synthesis was carried out on a 1.0 micromole scale according to protocols specified by the reagents' manufacturer. After complete cleavage and deprotection, the DNA products were precipitated by adding 250 μL 3 M NaCl and 5.0 mL cold ethanol, followed by incubation in a freezer at -20 °C. Afterwards, the precipitated DNA was collected through centrifugation, re-dissolved in 500 μL of 0.2 M triethylamine acetate (TEAA Glen Research Corp.), and then purified via HPLC (ProStar, Varian) with a C-18 reversed-phase column (Alltech, 5 μm, 250 mm × 4.6 mm). The collected eluent was dried and subjected to detritylation by dissolving and incubating in 200 μL 80% acetic acid for 20 minutes. The detritylated DNA was mixed with 400 μL ethanol and vacuum dried. Finally, the DNA concentrations were characterized with a Cary Bio-300UV spectrometer (Varian) using the absorbance of DNA at 260 nm.

Aligner-mediated cleavage. Both polyacrylamide gel electrophoresis (PAGE) and real-time fluorescence were used to characterize aligner-mediated cleavage. Using Nt.BstNBI as an example, a solution containing either 0.1 μM DA or 0.1 μM target sequence (or both) in 1× NEBuffer 3.1 (100 mM NaCl, 50 mM Tris-HCl, 10 mM $MgCl_2$, 100 μg/ml BSA, pH 7.9 @25 °C) with/without 10 U Nt.BstNBI was first incubated at 55 °C for 1 h and then transferred to 80 °C for 20 min to inactivate the enzyme. Afterwards, 10 μL of each resultant were loaded onto 15% denaturing polyacrylamide gel containing 8 M urea and subjected to 100 V constant voltage in 0.5× TBE buffer (45 mM Tris, 45 mM borate, 1 mM EDTA, pH 8.3) for 60 min at room temperature. The gel was then analyzed on a Typhoon FLA 7000

scanner (GE Healthcare Life Sciences) or a Maestro Ex IN-VIVO Imaging System (CRI). For real-time fluorescence measurement, a 24-μL solution containing either 0.1 μM DA or 0.1 μM target sequence (or both) in 1× NEBuffer 3.1 was first incubated on an iQ5 real-time PCR system (Bio-Rad) at 55 °C for 5-10 minutes, with the fluorescence monitored at 1 min intervals. Then, 1 μL of Nt.BstNBI (10 U) or H_2O was added promptly. The incubation and fluorescence measurements were continued for at least 60 min. For the sake of comparison, the original fluorescence data were corrected forthe background signals measured in the first 5-10 minutes before plotting the ultimate curves. For endonuclease Nt.AlwI, AlwI and MlyI, similar experiments were carried out in 1× CutSmart Buffer (50 mM Potassium Acetate, 20 mM Tris-acetate, 10 mM Magnesium Acetate, 100 μg/ml BSA, pH 7.9 @25 °C) at 37 °C.

For cleavage of the plasmid, a solution of 1 μg *pKD3* was first incubated at 95 °C for 5 min and fast-cooled on ice, followed by addition of 2 μM DNA Aligner (DA-16, Table S1), 10 U Nt.BstNBI and 1× NEBuffer 3.1. The mixture was incubated at 55 °C for 6 h. After deactivation at 80 °C for 20 min, the mixture was loaded onto 1% agarose gel and subjected to 90 V constant voltage in 1× TAE buffer (40 mM Tris, 20 mM acetic acid, 1 mM EDTA, pH 8.3) for 60 min at room temperature. The gel was then analyzed on a Maestro Ex IN-VIVO Imaging System (CRI).

AMC-based isothermal exponential amplification. First, a stock solution of 100 nM forward aligner (FA) and primer (FP), 100 nM reverse aligner (RA) and primer (RP) (see detailed sequences in Table S2), 6 U Nt.BstNBI, 0.8 U Bst 2.0 WarmStart DNA polymerase, 0.4 mM dNTPs, 0.5× SYBR Green I, 1× reaction buffer (pH 8.0, 100 mM NaCl, 50 mM Tris-HCl, 4 mM $MgCl_2$ and 100 μg/ml BSA) was freshly prepared on ice. The final reaction mixture was obtained by distributing 22.5 μL stock solution and 2.5 μL target DNA of varying concentrations into 8-strip PCR tubes. After vortexing and centrifuging, these PCR tubes were immediately transferred onto a Bio-Rad CFX96 real-time PCR system and incubated at 55 °C. The fluorescence of SYBR Green I was measured at 1 min intervals. Afterwards, the amplification products were loaded onto a 12% polyacrylamide gel immersed in 0.5× TBE buffer, followed by application of 120 V constant voltage for 50 min at room temperature. After staining with SYBR Green I, the PAGE gel was imaged on a CRI Maestro Ex IN-VIVO Imaging System.

Amplification of plasmid *pUC57*. The supercoiled plasmid *pUC57* was chosen to verify the applicability of AMC-SDA for double-stranded real sample. The target sequence of interest lies between the 1226th and 1339th base, with a common reverse aligner/primer pair (RA-4/RP-4) and varying forward counterparts (FA-4/FP-4, FA-5/FP-5 and FA-6/FP-6) to amplify three fragments of distinct length (see sequences listed in Table S2). Except for the general procedure described above, a two-step amplification was also tested. First, a solution of 1× reaction buffer, 0.4 mM dNTPs, 6 U Nt.BstNBI, 0.8 U Bst 2.0 WarmStart DNA polymerase and plasmid *pUC57* of varying concentration was incubated at 55

°C for 30 minutes on a Bio-Rad CFX96 real-time PCR system. Then, the forward and reverse aligners/primers, as well as SYBR Green I, were added. The final reaction mixture was kept at 55 °C for 60 more min, and the fluorescence of SYBR Green I was measured at 1 min intervals. The PAGE analysis followed the same procedure as that described above.

Detection of HBV DNA in real samples. To evaluate the practicality of AMC-SDA for real samples, the detection of HBV DNA in clinical serum specimens was performed. The target sequence of interest lies between the 1570th and 1650th base of HBV DNA, and the designed forward and reverse aligners/primers are FA-7/FP-7 and RA-7/RP-7, respectively (see sequences in Table S2). The experimental procedures were similar to those used in the amplification of plasmid *pUC*57. First, the sensitivity of AMC-SDA for HBV DNA was examined by detecting the HBV positive serum standards. Then, seven clinical serum specimens from suspicious HBV patients were tested, with four of them confirmed HBV positive and three HBV negative. DNA was extracted from these specimens using RNA/DNA Extraction/Purification Kits (DAAN Gene Co., Ltd.), transferred to PBS saline buffer, and subjected to AMC-SDA detection. These specimens were also detected using HBV TaqMan qPCR diagnosis kits (approved by the SFDA of China) according to the manufacturer's instructions.

Supporting Scheme and Figures

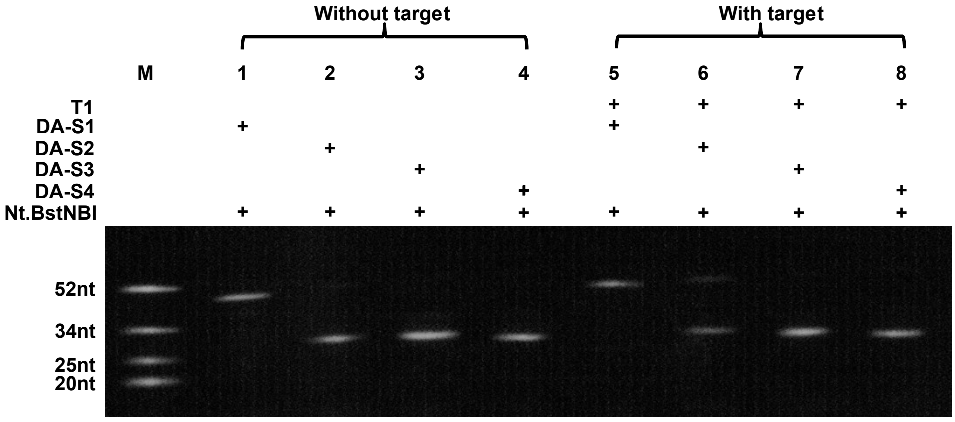

Fig. S1. Denaturing PAGE image of FAM-labeled DNA aligners (DA-S1～DA-S4, containing 1-4 A-T pairs beyond the recognition site, respectively; see Table S1 for sequence information) after incubation with Nt.BstNBI at 55 °C in the presence or absence of target DNA (T-1). The results show that two or more base pairs beyond the recognition site will lead to the digestion of DNA aligner with or without target DNA.

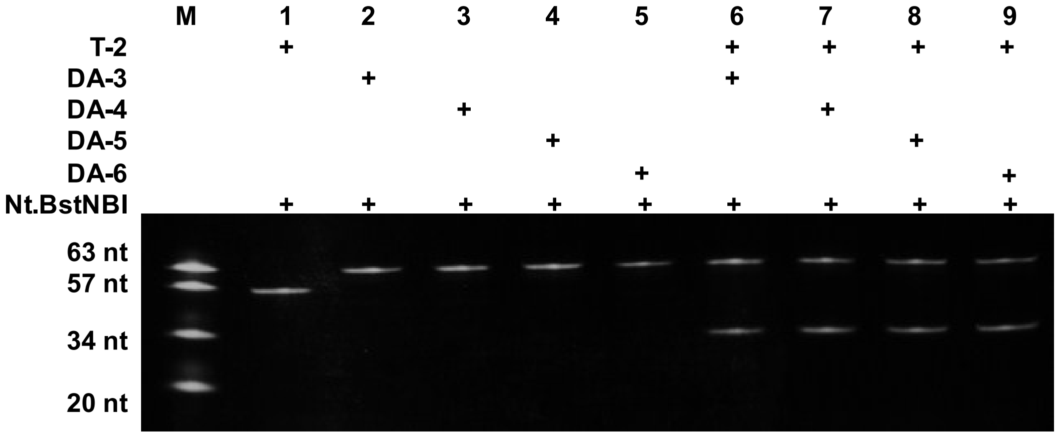

Fig. S2. Denaturing PAGE image of fluorescently labeled DNA aligners and target sequence (see Table S1 for detailed information) after incubation with Nt.BstNBI at 55 °C. The results show that aligner-mediated cleavage exclusively occurs on target DNA and that the conformational changes of DNA structure have little effect on it.

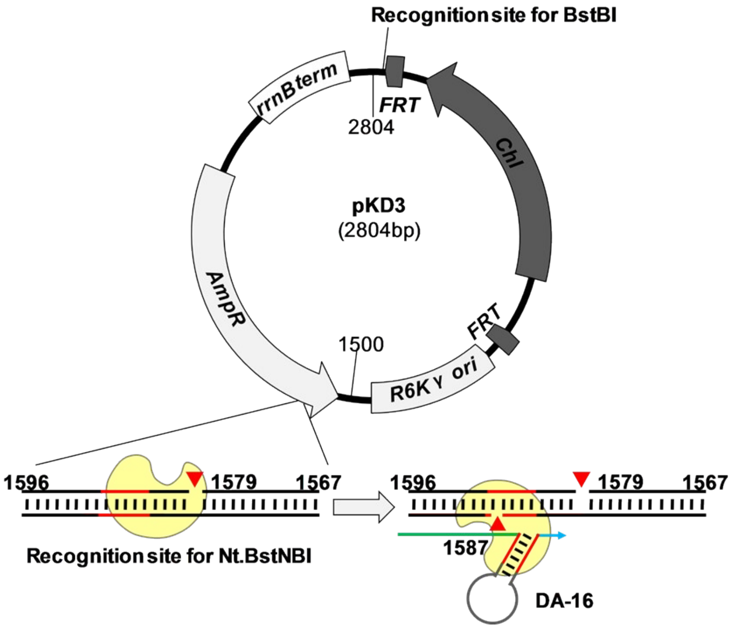

Fig. S3. Schematic illustration of plasmid *pKD3* cleavage. Plasmid *pKD3* contains each recognition sequence for BstBI (TTCGAA) and Nt.BstNBI (GAGTC), respectively. The former is a restriction endonuclease, while the latter is a nicking endonuclease. Thus, plasmid *pKD3* was cleaved on both strands by BstBI to form a linearized format (Lin), but nicked on only one strand by Nt.BstNBI to form an open circular format (OC). DNA aligner (DA-16) was devised to make a break eight bases away from the nicking site on the opposite strand. As a result, *pKD3* was linearized by Nt.BstNBI in the presence of DA-16, which showed a gel band similar to that of the double-strand cleavage by BstBI.

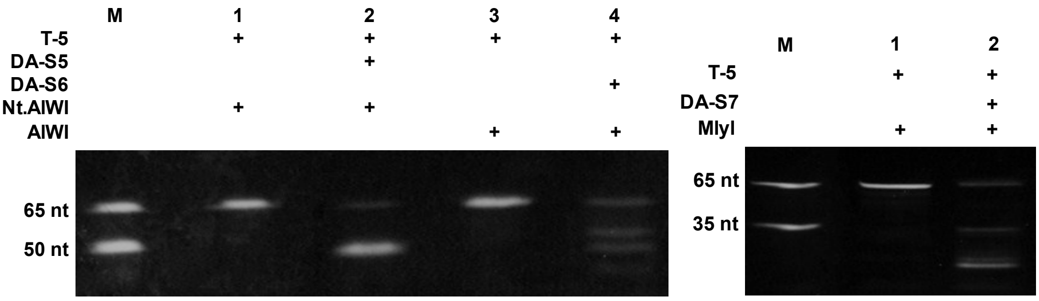

Fig. S4. Denaturing PAGE image of fluorescently labeled target sequence (see Table S1 for detailed information) after incubation with Nt.AlwI/AlwI (Left) and MlyI (Right) at 37 °C in the presence/absence of DNA aligner. The results show that all three endonucleases can effectively cleave target DNA with the help of DNA aligner DA-S5, DA-S6 and DA-S7, respectively. Given that AlwI and MlyI are double-strand cleaving enzymes, they should cut both target sequence and DNA aligner on Y-shaped structure; therefore, DA-S6 and DA-S7 were partly phosphorothioated (see Table S1 for detailed information). The results also show distinct cleavage patterns when using these two enzymes; however, the actual underlying mechanism remains to be investigated.

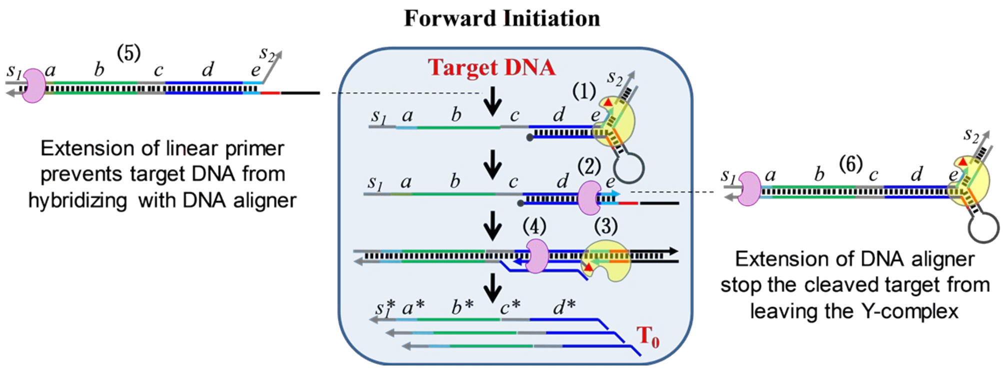

Fig. S5. The main function of 3'-termination is to prevent unexpected extensions of aligner/primer along target DNA. Taking forward initiation as an example, the extension of linear primer along target DNA before cleavage (Step 5) will impede target DNA from hybridizing with DNA aligner and thus prevent aligner-mediated cleavage (Step 1). On the other hand, the extension of DNA aligner (Step 6) will stop the cleaved target from leaving the Y-complex, preventing polymerase-catalyzed extension (Step 2). Both processes will result in a failed amplification.

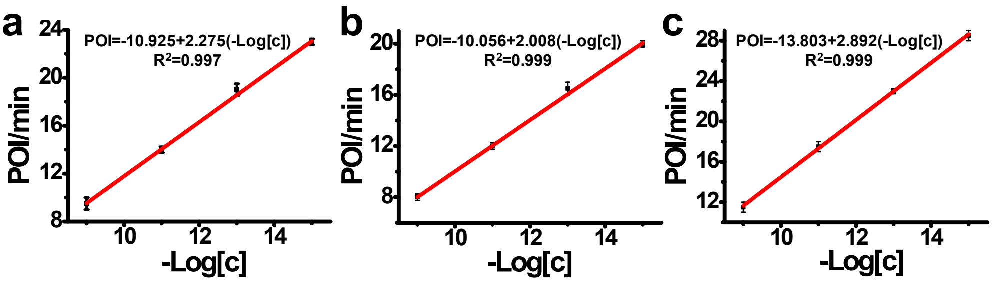

Fig. S6. The linear relationship between the POI value and Log[c] for TA-1, HIV Gag gene and HBV S gene (corresponding to Fig. 3c, 4a and 4b, respectively). [c] represents the concentration of target DNA.

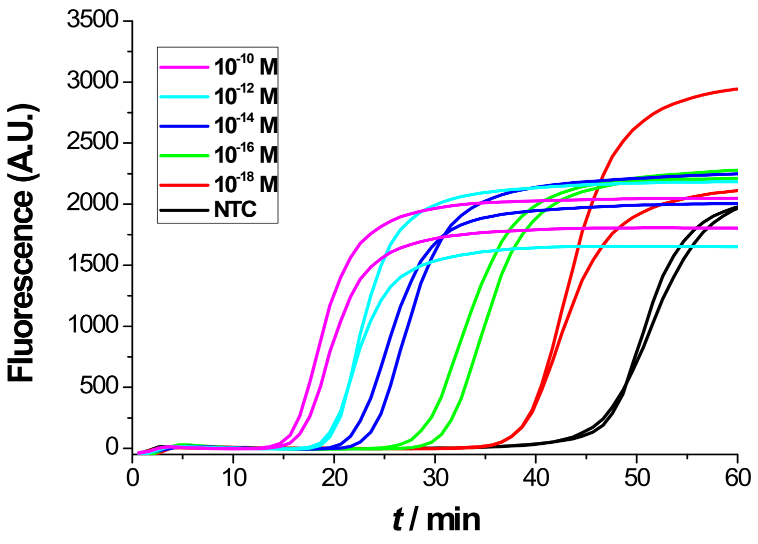

Fig. S7. Real-time fluorescence of SYBR Green I when amplifying different concentrations of target sequences (TA-1). Aligners/primers with high labeling efficiency of 3'-terminator will result in a lower LOD, down to 1 aM. The aligners/primers used here are RA-1/RP-1 and FA-1/FP-1 (see Table S2 for sequence information).

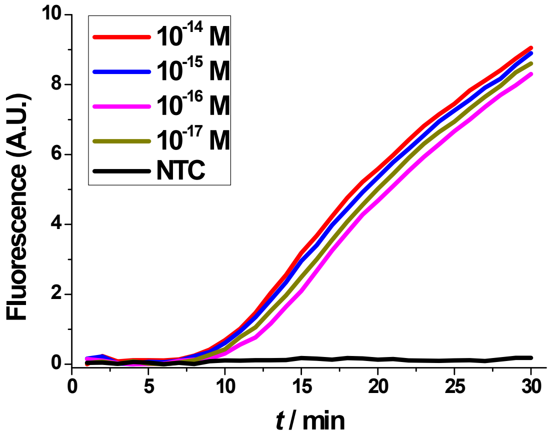

Fig. S8. Real-time fluorescence when using FAM- and Dabcyl-labeled molecular beacon (MB-S1) to monitor the amplification of target sequences (TA-S1). Although accurate quantification of target DNA is difficult, it could readily sense the existence of target DNA, even at a very low concentration. This experiment was carried out on an ABI 7900 Real-time PCR system using RA-1/RP-1 and FA-1/FP-1 as aligners/primers (see Table S2 for sequence information). NTC represents the no-target control.

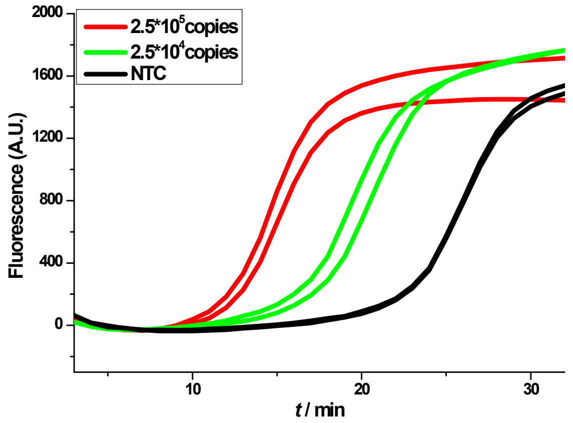

Fig. S9. Real-time fluorescence increase of SYBR Green I when amplifying different concentrations of HBV positive serum standards. The aligners/primers used here are RA-7/RP-7 and FA-7/FP-7 (see Table S2 for sequence information). NTC represents the no-target control.

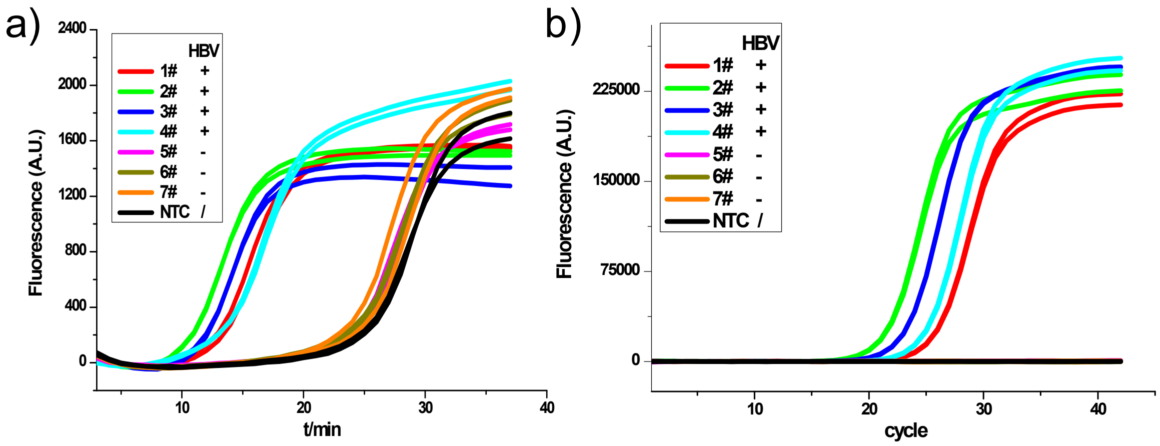

Fig. S10. a) Real-time fluorescence of AMC-SDA when amplifying DNA extracted from clinical serum specimens of suspicious HBV patients. The aligners/primers used here are RA-7/RP-7 and FA-7/FP-7 (see Table S2 for sequence information). NTC represents the no-target control. b) Real-time fluorescence of TaqMan qPCR for the same specimens. AMC-SDA and qPCR were performed on Bio-Rad CFX96 real-time PCR system and ABI 7900 rea-time PCR system, respectively.

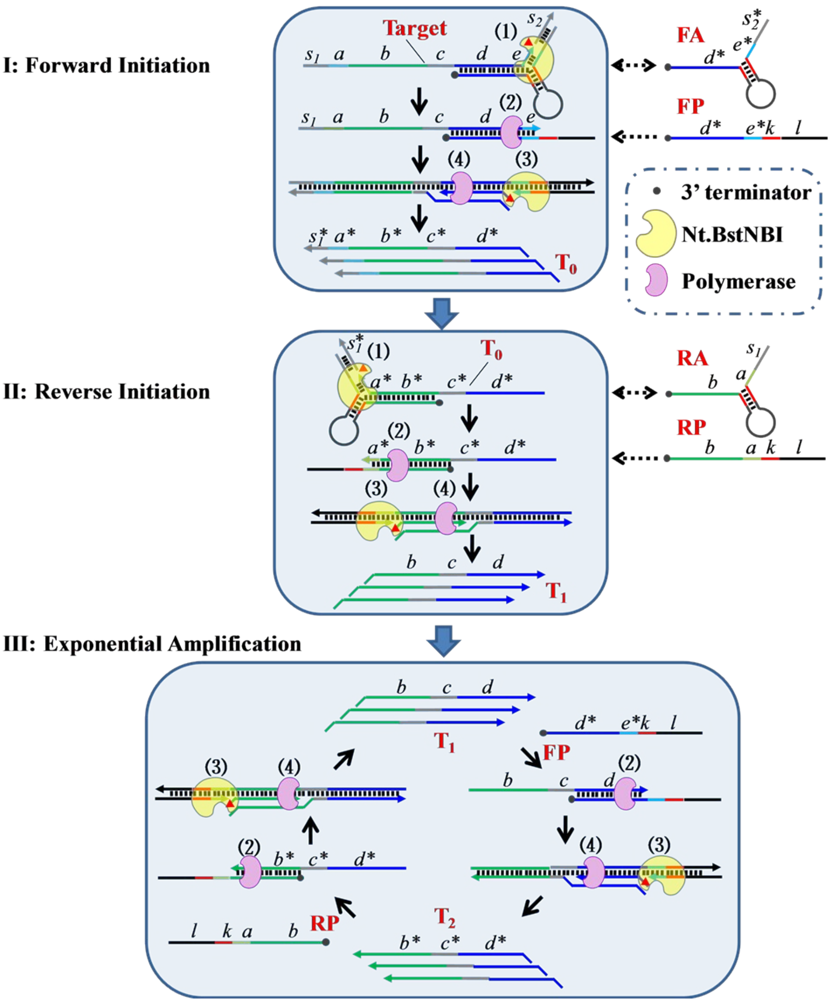

Scheme S1. Schematic illustration of AMC-SDA. (Step 1) Target DNA, or its antisense sequence, hybridizes with DNA aligner and is cleaved by Nt.BstNBI through AMC. (Step 2) The cleaved sequence with a definite 3'-end binds a linear primer, followed by polymerase-catalyzed extension along this linear primer to generate a complete double-stranded recognition site. (Step 3) Nt.BstNBI binds to the newly formed recognition site and makes a nick four bases downstream. (Step 4) Polymerase catalyzes the extension from the nicking site, displacing the previous strand. Step 3 and Step 4 can be repeated multiple times.

Table S1. Sequences used in AMC studies.[a]

Name	Sequence (5'-3')	Figure
T-1	5'-FAM-ATAATACTGCGTCTTGGTTCACAGC-3'	Fig. 1a
DA-1	5'-TGTGAA*GACTC*ACGTTTTTCGT*GAGTC*CCAAGACGCAGTATTAT-FAM-3'	
DA-2	5'-TGTGAAT*GACTC*ACGTTTTTCGT*GAGTC*ACCAAGACGCAGTATTAT-FAM-3'	
T-2	5'- CCAATAATACTGCGTCTTGGTTCACAGCTCTCACTCAGCATGGCAAGGAGGAACTTA-FAM-3'	Fig. 1b<br>Fig. S2
DA-3	5'-TGCTGAGTGAGAGCTGTGAAT*GACTC*ACGTTTTTCGT*GAGTC*ACCAAGACGCAAAAAAAAAAA-FAM-3'	
DA-4	5'-TGCTGAGTGAGAGCTGTGAAT*GACTC*ACGTTTTTCGT*GAGTC*ACCAAGAAAAAAAAAAAAAA-FAM-3'	
DA-5	5'-AAAAAAAAAAAAAATGTGAAT*GACTC*ACGTTTTTCGT*GAGTC*ACCAAGACGCAGTATTATTGG-FAM-3'	
DA-6	5'-AAAAAAAAAAGAGCTGTGAAT*GACTC*ACGTTTTTCGT*GAGTC*ACCAAGACGCAGTATTATTGG-FAM-3'	
T-3	5'-CGTCGTATTGGTTCTCTGCTC-3'	Fig. 1c
T-4	5'-Dabcyl-CGTCGTATTGGTTCTCTGCTC-FAM-3'	
DA-7	5'-GAGCAGAGAACCT*GACTC*ACGTTTTTCGT*GAGTC*AAATACGACG-3'	
DA-8	5'-Dabcyl-GAGCAGAGAACCT*GACTC*ACGTTTTTCGT*GAGTC*AAATACGACG-FAM-3'	
T-5	5'-GCTCATCACTGCACA CTCCTGTAAGGTTTC ACACATCTAAGGTCG TATTGCTCCTCTGGTCTCTC-FAM-3'	Fig. 2a
DA-9	5'-GGAGCAATACGAT*GACTC*ACGTTTTTCGT*GAGTC*ACCTTAGATGTGT-3'	
DA-10	5'-TAGATGTGTGAAT*GACTC*ACGTTTTTCGT*GAGTC*AACCTTACAGGAG-3'	
DA-11	5'-TTACAGGAGTGTT*GACTC*ACGTTTTTCGT*GAGTC*AGCAGTGATGAGC-3'	
T-6	5'-Dabcyl-CGTCGTATTGGTTCTCTGCTC-FAM-3'	Fig. 2b
T-7	5'-CGTCGTATTGG(Dabcyl-T)TCTCTGCTC-FAM-3'	
T-8	5'-CGTCGTATTGGT(Dabcyl-T)CTCTGCTC-FAM-3'	
T-9	5'-CGTCGTATTGGTTC(Dabcyl-T)CTGCTC-FAM-3'	
DA-12	5'-GAGCAGAGAACCT*GACTC*ACGTTTTTCGT*GAGTC*AAATACGACG-3'	
DA-13	5'-GAGCAGAGAACT*GACTC*ACGTTTTTCGT*GAGTC*ACAATACGACG-3'	
DA-14	5'-GAGCAGAGAAT*GACTC*ACGTTTTTCGT*GAGTC*ACCAATACGACG-3'	
DA-15	5'-GAGCAGAGAT*GACTC*ACGTTTTTCGT*GAGTC*AACCAATACGACG-3'	
DA-16	5'-CGTATCGTAGTTATCTACACGACGGGGAGTT*GACTC*ACGTTTTTCGT*GAGTC*AGTC-3'	Fig. 2c
DA-S1	5'- FAM-TGTGAAT*GACTC*ACGTTTTTCGT*GAGTC*ACCAAGACGCAGTATTAT-3'	Fig. S1
DA-S2	5'- FAM-TGTGAATT*GACTC*ACGTTTTTCGT*GAGTC*AACCAAGACGCAGTATTAT-3'	
DA-S3	5'- FAM-TGTGAATTT*GACTC*ACGTTTTTCGT*GAGTC*AAACCAAGACGCAGTATTAT-3'	
DA-S4	5'- FAM-TGTGAATTTT*GACTC*ACGTTTTTCGT*GAGTC*AAAACCAAGACGCAGTATTAT-3'	
DA-S5	5'-TTACAGGAGTGTT*GATCC*ACGTTTTTCGT*GGATC*AGCAGTGATGAGC-3'	Fig. S4
DA-S6[b]	5'-TTACAGG*A*G*TGTT*GATCC*ACGTTTTTCGT*GGATC*AGC*A*G*TGATGAGC-3'	
DA-S7[b]	5'-TACGACCTTAGATGT*G*T*GAAT*GACTC*ACGTTTTTCGT*GAGTC*AACC*T*T*ACAGGAGTGTGC-3'	

[a] The underlined bases represent the basic sequences of DNA aligners' stem-loop structure, among which the italicized red bases are the recognition sites of REases/NEases, and the purple bases indicate the extra AT base pairs beyond the recognition sequence. The complementary parts between target DNA and DNA aligner share the same color (green, light blue, orange and brown grey). [b] The asterisks indicate the sites of phosphorothioation.

Table S2. Sequences used in AMC-SDA studies.[a]

Name	Sequence (5'-3')	Source	Figure
TA-1	5'-GCTCATCACTGCACACTCCTGTAAGGTTTCACACATCTAACGTCGTATTGCTCCTCTGGTCTCTC-3'	Artificial sequence	Fig. 3 and Fig. S6
TA-2	5'-GCTCATCACTGCACACTCCTGTAAGGTTTCACACTAACCATCCCATCATCTCATCTAACGTCGTATTGCTCCTCTGGTCTCTC-3'		
TA-3	5'-GCTCATCACTGCACACTCCTGTAAGGTTTCACACTAACCATCACTAACCATCCCATCATCTCCCATCATCTCATCTAACGTCGTATTGCTCCTCTGGTCTCTC-3'		
TA-1-1	5'-GCTCATCACTGCACACTCCTGTAAGGTTTCACACATCTAACGTCGTATTGCTC*G*TCTGGTCTCTC-3'		
TA-1-2	5'-GCTCATCACTGCACACTCCTGTAAGGTTTCACACATCTAACGTCGTATTGCT*G*CTCTGGTCTCTC-3'		
TA-1-3	5'-GCTCATCACTGCACACTCCTGTAAGGTTTCACACATCTAACGTCGTATTGC*A*CCTCTGGTCTCTC-3'		
TA-1-4	5'-GCTCATCACTGCACACTCCTGTAAGGTTTCACACATCTAACGTCGTATTG*G*TCCTCTGGTCTCTC-3'		
Non-target	5'-TATCGCCACTGGCAGCAGCCACTGGTAACATGGTATCTGCGCTCTGCTGAAGCCAGTTACCT-3'		
RA-1	5'-GCTCATCACTGCACT*GACTC*ACGTTTTTCGT*GAGTC*AACTCCTGTAAG-inverted dT-3'		
RP-1	5'-ATGCATGCAT*GAGTC*GCACACTCCTGTAAGGT-inverted dT-3'		
FA-1	5'-GAGAGACCAGAGGAT*GACTC*ACGTTTTTCGT*GAGTC*AGCAATACGAC-inverted dT-3'		
FP-1	5'-ATGCATGCAT*GAGTC*AGGAGCAATACGACG-inverted dT-3'		
TA-S1	5'-GCTCATCACTGCACACTCCTGTAAGGTTTCAAAT*CTAACCATCCCATCATCT*TAATAACGTCGTATTGCTCCTCTGGTCTCTC-3'		Fig. S7
MB-S1	5'-FAM-CGTCAC*AGATGATGGGATGGTTAG*GTGACG-Dabcyl-3'		
TA-4	5'-CAGCATTATCAGAAGGAGCCACCCCACAAGATTTAAACACCATGCTAAACACAGTGGGGGGACATCAAGCAGCCATGC-3'	*HIV Gag Gene*[9]	Fig. 4a
RA-2	5'-CAGCATTATCAGAAGGT*GACTC*ACGTTTTTCGT*GAGTC*AAGCCACCCCAC-inverted dT-3'		
RP-2	5'-ATGCATGCAT*GAGTC*AAGGAGCCACCCCACA-inverted dT-3'		
FA-2	5'-GCATGGCTGCTTGATGT*GACTC*ACGTTTTTCGT*GAGTC*ATCCCCCCACTG-inverted dT-3'		
FP-2	5'-ATGCATGCAT*GAGTC*GATGTCCCCCCACTGT-inverted dT-3'		

TA-5	5'-GTCCTGGCCAAAATTCGCAGTCCCCAACCTCCAATCACTCACCAACCTCTTGTCCTCCAATTTGTCCTGGCTATCGCTGGATGTGTCTGCGG-3'	*HBV S Gene*[10]	Fig. 4b
RA-3	5'-GTCCTGGCCAAAATT*GACTC*ACGTTTTTCGT*GAGTC*ATCGCAGTCCCC-inverted dT-3'		
RP-3	5'-ATGCATGCAT*GAGTC*AAATTCGCAGTCCCCA-inverted dT-3'		
FA-3	5'-CCGCAGACACATCCT*GACTC*ACGTTTTTCGT*GAGTC*AAGCGATAGCCA-inverted dT-3'		
FP-3	5'-ATGCATGCAT*GAGTC*GTCCAGCGATAGCCAG-inverted dT-3'		
RA-4	5'-TATCGCCACTGGCAGT*GACTC*ACGTTTTTCGT*GAGTC*ACAGCCACTGGT-inverted dT-3'	Plasmid *pUC57* (No. 1226-1339 base)	Fig. 4c-e
RP-4	5'-ATGCATGCAT*GAGTC*GCAGCAGCCACTGGT-inverted dT-3'		
FA-4	5'-AGGCCACCACTTCAAT*GACTC*ACGTTTTTCGT*GAGTC*AGAACTCTGTAG-inverted dT-3'		
FP-4	5'-ATGCATGCAT*GAGTC*ACAAGAACTCTGTAGCA-inverted dT-3'		
FA-5	5'-GTAGCCGTAGTTAGGCT*GACTC*ACGTTTTTCGT*GAGTC*ACACCACTTCAAGA-inverted dT-3'		
FP-5	5'-ATGCATGCAT*GAGTC*AGGCCACCACTTCAAGA-inverted dT-3'		
FA-6	5'-ATACTGTTCTTCTAGT*GACTC*ACGTTTTTCGT*GAGTC*ATGTAGCCGTAG-inverted dT-3'		
FP-6	5'-ATGCATGCAT*GAGTC*ATAGTGTAGCCGTAGTT-inverted dT-3'		
RA-7	5'-AGACCTTGGGCAGGTTCCT*GACTC*ACGTTTTTCGT*GAGTC*ACC-inverted dT-3'	HBV specimen (No. 1570-1650 base)	Fig. S8 and S9
RP-7	5'-ATGCATGCAT*GAGTC*TCCGGTGGGCGTTCA-inverted dT-3'		
FA-7	5'-CCGGACCGTGTGCACTTCT*GACTC*ACGTTTTTCGT*GAGTC*ACT-inverted dT-3'		
FP-7	5'-ATGCATGCAT*GAGTC*TTCGCTTCACCTCTG-inverted dT-3'		

[a] The solid and dashed underlined bases represent the basic sequences of DNA aligners' stem-loop structure and linear primers' 5'-end, respectively. The italicized underlined red bases in TA-1-1~TA-1-4 indicate the mismatched bases compared with target DNA (TA-1). The complementary parts between target DNA and DNA aligner/primer share the same color (green, light blue, orange and brown grey).

Table S3. The comparison of AMC-SDA with other amplification methods.

Method	Detection limit	Dynamic range	Time	Complexity	Ref.
Stem-loop RT-PCR	2×10^{-17} M	7	1 h	Stringent temperature control	1
LAMP	0.4×10^{-18} M	/	1 h	Complicated primer-design	2
PG-RCA	5×10^{-17} M	7	>2.5 h	Multi-steps, additional ligation process	3
HDA	10^{3} copies	5	～1.5 h	4～5 proteins/enzymes	4
D-RCA	4×10^{-14} M	8	>6 h	Multi-steps, additional ligation process	5
RPA	10 copies	4	1 h	4～5 proteins/enzymes	6
BAD-AMP	5×10^{10} copies	4	40 min	One-step	7
Branched-RCA	8.5×10^{-13} M	3	>6 h	Multi-steps, additional ligation process	8
AMC-SDA	3×10^{-17} M[a] (～10^{2} copies)	7-8	～0.5 h	One-step, Easy primer-design	This work

[a]The detection limit was calculated to be 3×10^{-17} M according to the 3 times the standard deviation of the blank.

References

1 C. F. Chen, D. A. Ridzon, A. J. Broomer, Z. H. Zhou, D. H. Lee, J. T. Nguyen, M. Barbisin, N. L. Xu, V. R. Mahuvakar, M. R. Andersen, K. Q. Lao, K. J. Livak and K. J. Guegler, *Nucleic Acids Res.*, 2005, **33**, e179.

2 T. Notomi, H. Okayama, H. Masubuchi, T. Yonekawa, K. Watanabe, N. Amino and T. Hase, *Nucleic Acids Res.*, 2000, **28**, e63.

3 T. Murakami, J. Sumaoka and M. Komiyama, *Nucleic Acids Res.*, 2009, **37**, e19.

4 M. Vincent, Y. Xu and H. Kong, *EMBO Rep.*, 2004, **5**, 795-800.

5 Y. T. Zhou, Q. Huang, J. M. Gao, J. X. Lu, X. Z. Shen and C. H. Fan, *Nucleic Acids Res.*, 2010, **38**, e156.

6 O. Piepenburg, C. H. Williams, D. L. Stemple and N. A. Armes, *Plos Biol.*, 2006, **4**, 1115-1121.

7 A. R. Connolly and M. Trau, Angew. Chem. Int. Ed., 2010, 49, 2720-2723.

8 Y. Q. Cheng, X. Zhang, Z. P. Li, X. X. Jiao, Y. C. Wang and Y. L. Zhang, *Angew. Chem*. 2009, **121**, 3318-3322.

9 Y. L. Zeng, X. G. Zhang, K. Nie, X. Ding, B. Z. Ring, L. Y. Xu, L. Dai, X. Y. Li, W. Ren, L. Shi and X. J. Ma, *Gene* 2014, **541**, 123-128.

10 Z. J. Cai, G. Q. Lou, T. Cai, J. Yang, N. P. Wu, *J. Clin. Virol.* 2011, **52**, 288-294.
